# The Serotonin Receptor 6 Antagonist Idalopirdine and Acetylcholinesterase Inhibitor Donepezil Have Synergistic Effects on Brain Activity—A Functional MRI Study in the Awake Rat

**DOI:** 10.3389/fphar.2017.00279

**Published:** 2017-06-12

**Authors:** Craig F. Ferris, Praveen Kulkarni, Jason R. Yee, Mark Nedelman, Inge E. M. de Jong

**Affiliations:** ^1^Department of Psychology, Center for Translational NeuroImaging, Northeastern UniversityBoston, MA, United States; ^2^Ekam ImagingBoston, MA, United States; ^3^Division of Neurodegeneration, H. Lundbeck A/SValby, Denmark

**Keywords:** serotonin, acetylcholine, BOLD fMRI, cognition, Alzheimer's disease

## Abstract

The 5-HT_6_ receptor is a promising target for cognitive disorders, in particular for Alzheimer's disease (AD) and other CNS disorders. The high-affinity and selective 5-HT_6_ receptor antagonist idalopirdine (Lu AE58054) is currently in development for mild-moderate AD as adjunct therapy to acetylcholinesterase inhibitors (AChEIs). We studied the effects of idalopirdine alone and in combination with the AChEI donepezil on brain activity using BOLD (Blood Oxygen Level Dependent) functional magnetic resonance imaging (fMRI) in the awake rat. Idalopirdine (2 mg/kg, i.v.) alone had a modest effect on brain activity, resulting in activation of eight brain regions at the peak response. Of these, the cholinergic diagonal band of Broca, the infralimbic cortex, the ventral pallidum, the nucleus accumbens shell, and the magnocellular preoptic area were shared with the effects of donepezil (0.3 mg/kg, i.v.). Donepezil alone activated 19 brain regions at the peak response, including several cortical regions, areas of the septo-hippocampal system and the serotonergic raphe nucleus. When idalopirdine and donepezil were combined, there was a robust stimulation pattern with activation of 36 brain regions spread across the extended-amygdala-, striato-pallidal, and septo-hippocampal networks as well as the cholinergic system. These findings indicate that, whilst idalopirdine and donepezil recruit a number of overlapping regions including one of the forebrain cholinergic nuclei, the synergistic effect of both compounds extends beyond the cholinergic system and the effects of donepezil alone toward recruitment of multiple neural circuits and neurotransmitter systems. These data provide new insight into the mechanisms via which idalopirdine might improve cognition in donepezil-treated AD patients.

## Introduction

During the last two decades, the primary approach to the symptomatic treatment of the cognitive decline in Alzheimer's disease (AD) has been represented by the acetylcholinesterase inhibitors (AChEIs), aiming to relieve the cholinergic deficit by blocking the breakdown of the neurotransmitter acetylcholine (ACh). Although AChEIs improve cognitive function in AD patients, the benefits are considered modest (Birks and Harvey, 2006; Raina et al., 2008; Tan et al., 2014). With the approval of memantine, an NMDA receptor antagonist addressing glutamatergic dysfunction in AD, a non-cholinergic medication became available which is also increasingly used in combination with AChEIs. However, the progressive cognitive decline in AD has fueled the continued search for novel cognitive enhancers that may provide symptomatic relief in the face of continued neuropathology.

The serotonergic system, in particular, has regained interest for the treatment of cognitive disorders, including AD. The 5-HT_6_ receptor provides a promising target for the treatment of cognitive disorders (Meneses et al., 2011; Ramirez, 2013). The near exclusive localization in the brain and the predominant expression in regions that mediate cognition (e.g., hippocampus, cortex, and striatum) have triggered a wealth of studies into the therapeutic potential of 5-HT_6_ receptor ligands (Burton et al., 2015; Calhoun et al., 2017). The procognitive properties of antagonists of the 5-HT_6_ receptor have since been well-documented in preclinical animal models (Mitchell and Neumaier, 2005; Fone, 2008; Arnt et al., 2010; Meneses et al., 2011) that have also suggested a potential benefit of combining a 5-HT_6_ receptor antagonist with an AChEI, as 5-HT_6_ receptor antagonism was shown to potentiate the neurochemical, electrophysiological, and procognitive effects of the AChEI donepezil (Marcos et al., 2008; Dawson, 2011; de Bruin et al., 2011). Several 5-HT_6_ receptor antagonists have now entered clinical development for the treatment of AD (Maher-Edwards et al., 2011; Wilkinson and Windfeld, 2014; Calhoun et al., 2017; Ferrero et al., 2017). Of these, idalopirdine (Lu AE58054), a high affinity (*Ki* = 0.83 nm) and selective 5-HT_6_ receptor antagonist (Arnt et al., 2010), is furthest advanced, and in phase III development for the treatment of mild to moderate AD as an adjunct therapy to AChEIs.

The mechanisms via which 5-HT_6_ receptor antagonism alone, and in combination with an AChEI, mediates pro-cognitive effects are not well-understood. Previously, we have demonstrated by *in vivo* electrophysiology and microdialysis that idalopirdine potentiates and prolongs the effects of donepezil on neuronal oscillations and extracellular levels of acetylcholine in the rat dorsal hippocampus and prefrontal cortex (Amat-Foraster et al., 2016; Herrik et al., 2016). Such potentiation of the effects of donepezil could contribute to the procognitive effects of idalopirdine observed in donepezil-treated AD patients. Further, studies also showed that idalopirdine monotherapy increases gamma oscillations and extracellular levels of monoamines and glutamate in the rat prefrontal cortex (Amat-Foraster et al., 2016; Mork et al., 2017), suggesting that the effects of idalopirdine extend beyond just amplification of the effects of donepezil. Indeed, 5-HT_6_ receptor antagonists have been shown to regulate multiple neurotransmitter systems (reviewed in: Dawson, 2011). The cellular localization of the receptor, on glutamatergic and GABAergic neurons as well as select populations of GABAergic interneurons (Helboe et al., 2015), suggests that the 5-HT_6_ receptor is well-positioned to regulate the balance between excitatory and inhibitory signaling, which may have a broad impact on brain activity beyond regions where the receptor is expressed.

To investigate which integrated neural circuits mediate the separate and combined effects of 5-HT_6_ receptor antagonism and AChE inhibition on general brain activity, we turned to the field of functional MRI (fMRI) in awake rodents (Ferris et al., 2011). Awake animal imaging has become an important tool in preclinical drug discovery (Borsook et al., 2006; Ferris et al., 2011; Haensel et al., 2015). Non-invasive fMRI provides a window to the brain making it possible to image changes in activity across distributed, integrated neural circuits with high temporal and spatial resolution. When combined with the use of 3D segmented, annotated, brain atlases, and computational analysis, it is possible to reconstruct distributed and integrated neural circuits or “finger prints” of brain activity. These finger prints may be used to characterize the activity and function of new psychotherapeutics in preclinical development and to study the neurobiology of integrated neural circuits controlling cognition and emotion. To this end, the present study investigates the separate and combined effects of 5-HT_6_ receptor antagonism and AChE inhibition on general brain activity. The imaging data show a pronounced synergistic effect between idalopirdine and donepezil that extends across several integrated neural circuits and different neurotransmitter systems.

## Methods

### Animals

A total of 48 male Sprague Dawley rats (Charles River Labs, MA USA) were enrolled for use in the study. At study initiation, the rats weighed between 275–350 g and were 3–4 months of age. The animals were housed in groups of 2 (cage size 30.5 × 43.2 × 17.8 cm). Animals were maintained in a room with a 12-h light/dark cycle (lights on at ~7:00 A.M.). Temperature was maintained at ~21°C. Rats were provided water through an automatic water distribution system (filtered to five microns). Food and water were available *ad libitum*. As part of an enrichment program, animals were provided nylabones, and sunflower seeds for foraging. Upon receipt, rats were acclimated for 5 days prior to use in the study. All rats were acquired and cared for in accordance with the guidelines published in the Guide for the Care and Use of Laboratory Animals (National Institutes of Health Publications No. 85–23, Revised 1985) and adhered to the National Institutes of Health and the American Association for Laboratory Animal Science guidelines. The protocols used in this study were approved by the Institutional Animal Care and Use Committee at the Northeastern University.

### Acclimation

To reduce the stress associated with head restraint, rats were acclimated to the restraining system (head holder and body tube) 1 week prior to their actual imaging session. The design of the restraining system (Animal Imaging Research, Holden, MA, USA) included a padded head support obviating the need for ear bars helping to reduce animal discomfort while minimizing motion artifact. Rats were briefly anesthetized (~5–7 min) with 2–3% isoflurane while being secured into the head holder. The forepaws were secured with tape. When fully conscious, the restraining system is placed into a black opaque box “mock scanner” for 60 min with a tape-recording of the MRI pulse sequence to simulate the bore of the magnet and the imaging protocol. Rats were acclimated for 5 consecutive days for 60 min each day, and randomly selected to be imaged within 7 days of the last acclimation day. The acclimation results in a significant decline in respiration, heart rate, motor movements, and plasma corticosterone when the first and last acclimation periods are compared (King et al., 2005). This reduction in autonomic and somatic measures of arousal and stress improve the signal resolution and quality of the images. Of the 48 rats, four continued to struggle to escape over the 5 day acclimation period and were excluded from the study. During imaging, an additional seven were omitted because of motion artifact that could not be corrected and one for electrical interference.

### Drug administration

Final group sizes following drug administration were: vehicle (*n* = 9), idalopirdine (*n* = 10), donepezil (*n* = 8), idalopirdine/donepezil (*n* = 9). All drugs were given i.v. through a tail vein catheter during the imaging session. Rats were briefly anesthetized with 2–3% isoflurane while being catheterized and secured into the head holder. Idalopirdine (Lu AE58054, Lundbeck) was dissolved in 5% HpBeta cyclodextrin in distilled water and evaluated at the dose of 2 mg/kg. Donepezil hydrochloride (Lundbeck) was dissolved in 5% HpBeta cyclodextrin in distilled water and evaluated at the dose of 0.3 mg/kg. The combination of idalopirdine (2 mg/kg) and donepezil (0.3 mg/kg) was also evaluated. The choice of doses in this single-dose pharmacological study was based on previous studies that demonstrated that these doses of idalopirdine and donepezil result in clinically relevant exposure of both compounds, high 5-HT6 receptor occupancy up to an hour after administration and synergistic pharmacological effects on neuronal activity and neurotransmitter release in both the cortex and hippocampus (Amat-Foraster et al., 2016; Herrik et al., 2016; Mork et al., 2017). The vehicle control was 5% HpBeta cyclodextrin in distilled water. All injections were given in a volume of 1 ml/kg.

### Image acquisition

Animals were scanned at 300 MHz using a quadrature transmit/receive volume coil built into the rat head holder and restraining system for awake animal imaging (Animal Imaging Research, Holden, MA, USA). A video of the rat preparation for imaging is available at www.youtube.com/watch?v=JQX1wgOV3K4. The design of the coil provided complete coverage of the brain from olfactory bulbs to brain stem. Experiments were conducted using a Bruker Biospec 7.0T/20-cm USR horizontal magnet (Bruker, Billerica, MA, USA) and a 20-G/cm magnetic field gradient insert (ID = 12 cm) capable of a 120-μs rise time. At the beginning of each imaging session, a high-resolution anatomical data set was collected using the RARE pulse sequence (22 slice; 1.0 mm; field of vision [FOV] 3.0 cm; 256 × 256; repetition time [TR] 2.5 s; echo time [TE] 12 ms; NEX (number of averages) 2; ca. 2.5 min acquisition time). Functional images were acquired using a multi-slice HASTE pulse sequence (Half Fourier Acquisition Single Shot Turbo Spin Echo). Bruker Paravision automatically finds the basic frequency, shims, and power requirements for 90° and 180° pulses and sets the receiver gain. A single scanning session acquired 22 slices, 1.0 mm thick, every 6.0 s (TR 6.0 s, TE 48 ms, FOV 3.0 cm, matrix size 96 × 96, NEX 1) repeated 500 times for a total time of 50 min. The in-plane pixel resolution is 312 μm^2^. Each scanning session was continuous for 50 min, starting with 50 baseline image acquisitions during 5 min prior to treatment, then drug presentation followed by 450 image acquisitions during the following 45 min.

**Figure 1:**
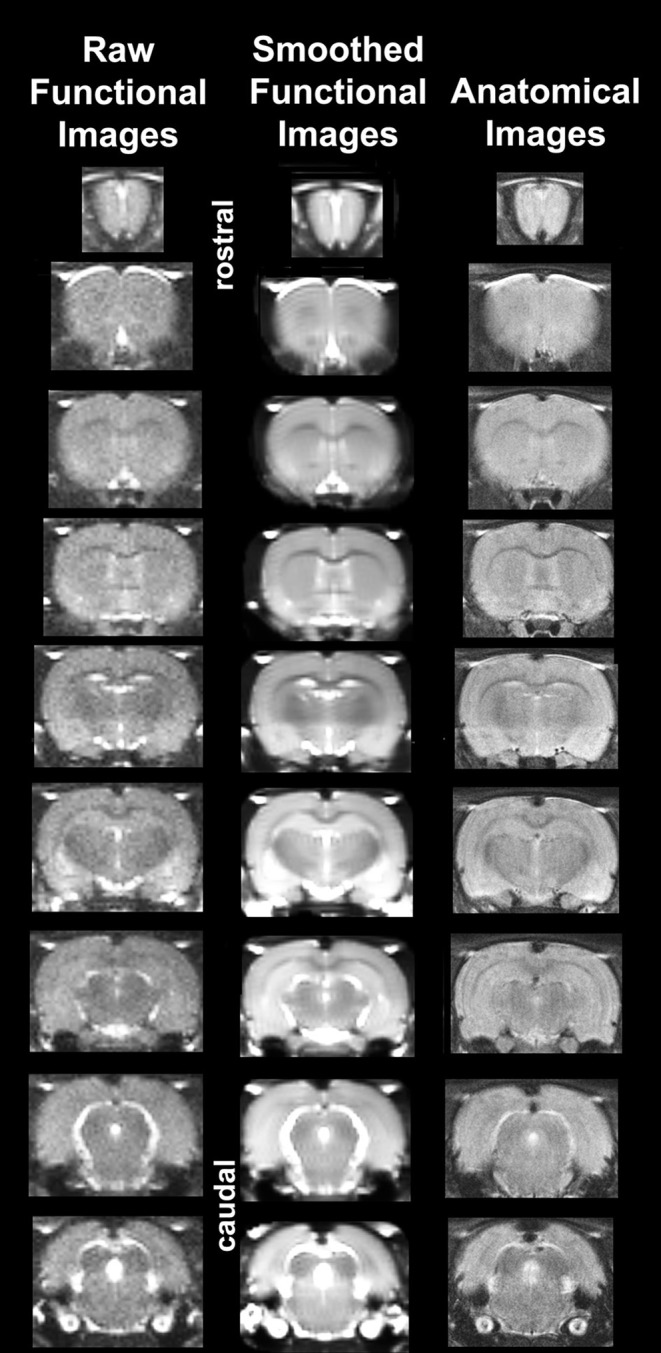
Neuroanatomical fidelity. Representative examples of brain images collected during a single imaging session using a multi-slice spin echo, RARE (rapid acquisition with relaxation enhancement) pulse sequence. The column on the right shows axial sections collected during the anatomical scan taken at the beginning of each imaging session using a data matrix of 256 × 256, 22 slices in a field of view of 3.0 cm. The column on the left shows the same images but collected for functional analysis using HASTE, a RARE pulse sequence modified for faster acquisition time. These images were acquired using the same field of view and slice anatomy but a larger data matrix of 96 × 96. The images in the middle column have been smoothed during pre-processing. Note the anatomical fidelity between the functional images and their original anatomical image. The absence of any distortion is necessary when registering the data to atlas to resolve 171 segmented brain areas.

It should also be emphasized that high neuroanatomical fidelity and spatial resolution are critical in identifying distributed neural circuits in any animal imaging study. Many brain areas in a segmented rat atlas have in-plane boundaries of <400 μm^2^ and may extend for over 1000 μm in the rostral/caudal plane. With the development of a segmented, annotated 3D MRI atlas for rats (Ekam Solutions, Boston, MA, USA) it is now possible to localize functional imaging data to precise 3D “volumes of interest” in clearly delineated brain areas. Therefore, it is critical that the functional images are a very accurate reconstruction of the original brain neuroanatomy as shown in Figure 1. To achieve this we used spin echo BOLD (Norris, 2012). The HASTE sequence, a spin-echo multislice pulse sequence, is insensitive to the artifacts of field inhomogeneity, susceptibility artifact, chemical shift, and other imaging distortions. The BOLD signal becomes a function of dynamic dephasing from diffusion of water at the level of the capillaries (Duong et al., 2003). Using spin echo BOLD the signal contrast with BOLD imaging is a function of T2 and not T2^*^ at high field strengths. The extravascular signal surrounding capillary beds and small vessels is more reflective of the metabolic changes in brain parenchyma than signal from large draining veins helping to improve the localization of the signal changes (Yacoub et al., 2007). The BOLD signal is linear and reproducible at stimulus intervals of 1 s (Zhang et al., 2009).

### Data analysis/statistics

Functional MRI data analysis included four primary steps: (1) preprocessing, including slice timing correction, co-registration, smoothing, and de-trending; (2) registration to rat brain atlas, followed by segmentation; (3) voxel-wise statistical analysis for each individual to identify voxels that experienced a signal change in relation to baseline; (4) group comparisons on the number of activated voxels per ROI and neural network.

#### Pre-processing

Preprocessing of functional scans was performed using in-house MATLAB® (The Mathworks, Inc, Natick MA.) software in combination with SPM8 (http://www.fil.ion.ucl.ac.uk/spm/) batch interface. Slice timing correction was done using SPM 8 algorithm (TR = 6 s, slice Order: Interleaved slices, bottom-up, 1:2:22, 2:2:22, Reference slice: middle). The mean image of all functional time points was computed and using this mean image, the data were roughly cropped slice by slice using a graphic user interface. This rough cropping insured improved motion correction without losing any voxels in the brain. Data was then co-registered to a mean functional image using SPM8's co-registrational code with the following parameters: Quality: 0.97, Smoothing: 0.35 mm, Separation: 0.5 mm. Gaussian smoothing was performed with a Full Width Half Max kernel width (FWHM) of 0.8 mm. Preprocessed functional files were then exported to Medical Image Visualization and Analysis (MIVA, EKAM Solutions, available upon request) for registration and segmentation.

#### Atlas registration and segmentation

Using MIVA, each subject was registered to a segmented rat brain atlas. Segmentation was performed using a one to one correlation to atlas voxels. While segmenting brain regions special care was taken to account for a partial volume effect as described in following ISMRM paper: http://cds.ismrm.org/ismrm-2005/Files/01565.pdf. The alignment process was facilitated by an interactive graphic user interface. The affine registration involved translation, rotation, and scaling in all three dimensions independently. The matrices that transformed the subject's anatomy to the atlas space were used to embed each slice within the atlas. The mean functional image (output from SPM) was used to register images to the rat atlas since the quality of the functional scans are structurally superior to those obtained using gradient echo (GRE) or echo planar imaging (EPI) sequences as noted above (see Figure 1). All transformed pixel locations of the anatomical images were tagged with regions in the segmented atlas creating a fully segmented representation of each subject.

#### Data analysis

Low frequency drift is a common problem in time series fMRI studies and contributes to signal variability. Instability in temperature regulation with high performance gradients is one potential source of the problem; however, physiological noise and head motion are also considered to be contributing factors for drift. The occurrence of false positive voxels is a concern particularly in the simple off-on activation paradigms used in these studies comparing the average baseline signal for a given voxel to its average post-stimulation signal. This effect may be more pronounced in longer imaging acquisition times as was utilized in this study design (~50 min scans). The data were detrended by calculating average drift form all segmented voxels and correcting individual voxels based on average drift. We assume drift to be linear for all the functional images. After segmentation and brain extraction global drift was computed by calculating average intensity of the whole brain for each repetition across the entire scan session. Slope of the data was computed with “polyfit” function in Matlab®; if the slope was more than predefined limit (±0.015% signal/ min), then each individual voxel was corrected for drift based on computed global slope. This strategy was adopted over voxel wise drift correction to avoid overcorrection due to activation in the ROI scans.

In voxel-based analysis, the BOLD % change of each independent voxel was computed in its original space. The signal was filtered for all subjects with a baseline threshold of 2% BOLD change to account for normal fluctuation of BOLD signal in the awake rat brain (Brevard et al., 2003). Statistical *t*-tests were performed on each voxel (ca. 15,000 in number) of each subject within their original coordinate system. The average signal intensity in each voxel of the first 5 min of baseline (acquisitions 1–50) was compared to minutes 15–25 (acquisitions 150–250), 25–35 (acquisitions 250–350), and 35–45 (acquisitions 350–450) post-treatment. *t*-test statistics with a 95% confidence level, two-tailed distributions, and heteroscedastic variance assumptions were applied. As a result of the multiple *t*-test analyses performed, a false-positive detection controlling mechanism was introduced (Genovese et al., 2002). This subsequent filter guaranteed that, on average, *p*-value of test statistics was below our cutoff of 0.05. A composite image of the whole brain representing the average of all subjects was constructed for each group for ROI analyses, allowing us to look at each ROI separately to determine the BOLD change and the number of activated voxels in each ROI. Volume of activation was compared across experiment groups using the non-parametric Kruskall-Wallis test statistic. Brain areas were considered statistically different between experiment groups when comparison produced *p* < 0.05.

Change in BOLD signal over time for each of the experimental conditions was analyzed using a repeated measures ANOVA followed by Dunnett's *post-hoc* test which controls for multiple comparisons of multiple treatment groups to a single control group (“Vehicle”) (Dunnett, 1955). *Post-hoc* analyses was confined to comparisons between minutes 15–25, 25–35, and 35–45 post injection for each drug treatment and vehicle to match the statistical comparisons used for the volume of activation data.

## Results

### Comparison of the treatments to vehicle

Tables 1–**3** are a selection from 171 brain regions and show only those brain regions that are significantly different in volume of activation (voxel number) between vehicle and drug treatments over the entire post treatment period (15–45 min). A full list of all 171 brain regions for all treatment comparisons can be found in the Supplementary Data (Supplementary Tables 1–6). Comparison of the acute effect of idalopirdine to vehicle on BOLD signal in 171 brain regions showed little to no significant changes within the first 15–35 min post-treatment. By 35–45 min, idalopirdine showed increased activity in only eight brain regions, three belonging to the mesencephalic dopaminergic system (ventral pallidum, accumbens shell, substantia nigra pars reticularis) and one to the basal forebrain cholinergic system (diagonal band of Broca, dBB). In addition, the infralimbic cortex, periaqueductal gray (PAG), medial preoptic area, and magnocellular preoptic nucleus were activated.

**Table 1:** fMRI BOLD response for idalopirdine compared to vehicle following a single administration.

**Vehicle vs. Idalopiridine, postive BOLD volume of activation**											
**15–25 min post-treatment**				**25–35 min post-treatment**				**35–45 min post-treatment**			
**Brain area**	**Veh**	**I**	***P*****-val**	**Brain area**	**Veh**	**I**	***P*****-val**	**Brain area**	**Veh**	**I**	***P*****-val**
Reuniens nucleus	0	2	0.073	**Ventral pallidum**	**0**	**5**	**0.022**	**Ventral pallidum**	**0**	**8**	**0.006**
Ventral pallidum	0	3	0.073	**Magnocellular preoptic nucleus**	**0**	**1**	**0.026**	**Accumbens shell**	**0**	**10**	**0.008**
Magnocellular preoptic nucleus	0	0	0.082	Secondary somatosensory ctx	4	32	0.056	**Diagonal band of Broca**	**0**	**7**	**0.008**
Medial pretectal area	0	0	0.082	Accumbens shell	0	6	0.069	**Medial preoptic area**	**1**	**8**	**0.013**
Substantia innominata	0	0	0.082	Diagonal band of Broca	0	4	0.092	**Infralimbic ctx**	**0**	**14**	**0.015**
Accumbens shell	0	4	0.102	Caudal piriform ctx	0	23	0.097	**Substantia nigra reticularis**	**6**	**17**	**0.033**
Medial septum	0	0	0.114	Supramammillary nucleus	0	1	0.111	**Periaqueductal gray thalamus**	**24**	**47**	**0.037**
Lateral preoptic area	0	3	0.137	Inferior colliculus	62	85	0.121	**Magnocellular preoptic nucleus**	**0**	**1**	**0.042**

*Shown are three sets of results each representing comparisons between vehicle (Veh, n = 9) and idalopirdine (I, n = 10) treatment groups at times 15–25 (left), 25–35 (middle), and 35–45 (right) min post treatment. The probability values presented on the far-right column were derived using a Kruskall-Wallis test statistic. Lists were generated to include all areas (out of a total list of 171 areas that comprise the rat MRI atlas) that differed significantly (p < 0.05) for the epoch in which we observed the most changes (i.e., the 35–45 min epoch). Areas are rank-ordered by p-value for visualization. Areas highlighted in bold differed significantly between treatment groups. Areas listed in gray show areas that come closest to threshold for statistical significance*.

When comparing the acute effect of donepezil to vehicle on BOLD signal, there were 19 brain regions that already showed significant activation within 15–25 min after treatment (Table 2). These areas included several limbic cortical areas (infralimbic, orbital, insular, frontal association, entorhinal), regions belonging to the septo-hippocampal system (medial septum, ventral dentate gyrus, entorhinal cortex), the ventral pallidum, and the serotonergic dorsal raphe nucleus. Later time points evaluated in this study (>25 min following administration) showed fewer brain regions significantly activated, one of them being the cholinergic dBB.

**Table 2:** fMRI BOLD response for donepezil compared to vehicle following a single administration.

**Vehicle vs. Donepezil, positive BOLD volume of activity**											
**15–25 min post-treatment**				**25–35 min post-treatment**				**35–45 min post-treatment**			
**Brain area**	**Veh**	**D**	***P*****-val**	**Brain area**	**Veh**	**D**	***P*****-val**	**Brain area**	**Veh**	**DON**	***P*****-val**
**Magnocellular preoptic nucleus**	**0**	**1**	**0.007**	**Accumbens shell**	**0**	**5**	**0.013**	**Raphe obscurus nucleus**	**3**	**0**	**0.007**
**Dorsal raphe**	**0**	**8**	**0.012**	**Infralimbic ctx**	**0**	**10**	**0.013**	**Infralimbic ctx**	**0**	**15**	**0.01**
**Medial septum**	**0**	**3**	**0.02**	**Medial septum**	**0**	**2**	**0.02**	**Ventral orbital ctx**	**0**	**7**	**0.016**
**Medial orbital ctx**	**0**	**16**	**0.022**	**Magnocellular preoptic nucleus**	**0**	**2**	**0.021**	**Diagonal band of Broca**	**0**	**3**	**0.017**
**Ventral pallidum**	**0**	**12**	**0.027**	**Anterior olfactory nucleus**	**0**	**17**	**0.025**	**Crus 2 of ansiform lobule**	**39**	**4**	**0.021**
**Medial geniculate**	**0**	**22**	**0.028**	**Lateral geniculate**	**3**	**12**	**0.046**	**8th cerebellar lobule**	**14**	**0**	**0.033**
**Insular ctx**	**14**	**107**	**0.03**	**Crus 2 of ansiform lobule**	**39**	**2**	**0.047**	**7th cerebellar lobule**	**15**	**0**	**0.04**
**Superior colliculus**	**29**	**112**	**0.03**	Ventral orbital ctx	0	4	0.06	Medial pretectal area	0	0	0.051
**Frontal association ctx**	**0**	**10**	**0.032**	Habenula nucleus	3	19	0.065	Accumbens shell	0	8	0.052
**Inferior colliculus**	**41**	**108**	**0.034**	Dorsomedial tegmental area	0	2	0.072	Gigantocellular reticular nucleus pons	33	11	0.054
**Primary somatosensory ctx upper lip**	**0**	**41**	**0.041**	Diagonal band of Broca	0	4	0.075	6th cerebellar lobule	46	16	0.054
**Dentate gyrus ventral**	**1**	**39**	**0.043**	Medial geniculate	1	14	0.076	Crus 1 of ansiform lobule	52	14	0.054
**Anterior olfactory nucleus**	**0**	**33**	**0.044**	Subiculum dorsal	6	26	0.083	Ventral pallidum	0	9	0.058
**External plexiform layer**	**23**	**56**	**0.047**	Entorhinal ctx	43	118	0.083	Dorsal raphe	0	3	0.059
**Dorsal paragigantocellularis nucleus**	**0**	**3**	**0.047**	Dorsal raphe	0	3	0.084	Magnocellular preoptic nucleus	0	1	0.064
**Ventral orbital ctx**	**0**	**4**	**0.048**	Periaqueductal gray thalamus	9	55	0.092	Paramedian lobule	38	12	0.065
**Entorhinal ctx**	**27**	**147**	**0.048**	Ventral pallidum	0	6	0.096	Pontine nuclei	34	18	0.068
**Lateral geniculate**	**2**	**19**	**0.049**	3rd cerebellar lobule	4	17	0.099	Neural lobe pituitary	6	3	0.07
**Intercalated amygdaloid nucleus**	**0**	**0**	**0.05**	Frontal association ctx	0	6	0.103	Inferior olivary complex	13	3	0.076

*Shown are three sets of results each representing comparisons between vehicle (Veh, n = 9) and donepezil (D, n = 8) treatment groups at times 15–25 (left), 25–35 (middle), and 35–45 (right) min post treatment. The probability values presented on the far-right column were derived using a Kruskall-Wallis test statistic. Lists were generated to include all areas (out of a total list of 171 areas that comprise the rat MRI atlas) that differed significantly (p < 0.05) for the epoch in which we observed the most changes (i.e., the 15–25 min epoch). Areas are rank-ordered by p-value for visualization. Areas highlighted in bold differed significantly between treatment groups. Areas listed in gray show areas that come closest to threshold for statistical significance*.

When comparing the acute effect of the combination of idalopirdine plus donepezil to vehicle a greater number of brain regions showed significant activation when compared to either of the treatments alone. The change in BOLD signal was most robust at 25–35 min after treatment, with activation of 36 brain regions (Table 3). The pattern of activation was spread across multiple neural circuits and signaling pathways, including most of the brain regions that were found activated by one or both treatments individually. In addition, it should be emphasized that the combination treatment activated more brain regions than the addition of those observed with each drug alone. Regions activated by the combination treatment included the cholinergic pendunculopontine tegmentum (PPT), Bed Nucleus of the Stria Terminals (BNST), habenula, anterior cingulate cortex, specific areas of the mesencephalic dopamine system (ventral tegmental area, nucleus accumbens core, prelimbic cortex), amygdala (medial and cortical), septum (triangular, lateral), thalamus (medial dorsal, ventromedial, lateral posterior, and parafascicular), hypothalamus (anterior, posterior, and premammillary), and olfactory system (granular, glomerular, and external plexiform layers of the olfactory bulb, anterior olfactory n., tenia tecta).

**Table 3:** fMRI BOLD response for idalopirdine plus donepezil compared to vehicle following a single administration.

**Vehicle vs. Donepezil and Idalopiridine, positive BOLD volume of activity**											
**15–25 min post-treatment**				**25–35 min post-treatment**				**35–45 min post-treatment**			
**Brain area**	**Veh**	**I/D**	***P*****-val**	**Brain area**	**Veh**	**I/D**	***P*****-val**	**Brain area**	**Veh**	**I/D**	***P*****-val**
**Magnocellular preoptic nucleus**	**0**	**2**	**0.012**	**Infralimbic ctx**	**0**	**39**	**0.006**	**Infralimbic ctx**	**0**	**28**	**0.002**
**Medial preoptic area**	**0**	**25**	**0.014**	**Granular cell layer**	**15**	**81**	**0.006**	**Medial orbital ctx**	**0**	**21**	**0.007**
**Medial amygdaloid nucleus**	**2**	**12**	**0.014**	**Accumbens shell**	**0**	**13**	**0.006**	**Triangular septal nucleus**	**3**	**10**	**0.011**
**Ventral pallidum**	**0**	**15**	**0.018**	**Anterior olfactory nucleus**	**0**	**33**	**0.007**	**Anterior olfactory nucleus**	**1**	**32**	**0.014**
**Median raphe nucleus**	**0**	**4**	**0.018**	**Lateral preoptic area**	**0**	**8**	**0.008**	**Accumbens shell**	**0**	**15**	**0.016**
**Anterior olfactory nucleus**	**0**	**32**	**0.019**	**Habenula nucleus**	**3**	**18**	**0.009**	**Entorhinal ctx**	**51**	**168**	**0.017**
**Ventral tegmental area**	**0**	**4**	**0.03**	**Ventral pallidum**	**0**	**5**	**0.009**	**Ventral pallidum**	**0**	**4**	**0.018**
**Posterior hypothalamic area**	**0**	**12**	**0.03**	**Triangular septal nucleus**	**0**	**9**	**0.012**	**Granular cell layer**	**36**	**75**	**0.019**
**Medial dorsal thalamic nucleus**	**0**	**9**	**0.031**	**Medial preoptic area**	**0**	**26**	**0.012**	**Medial preoptic area**	**1**	**22**	**0.022**
**Pedunculopontine tegmental area**	**0**	**4**	**0.033**	**3rd cerebellar lobule**	**4**	**22**	**0.013**	**Tenia tecta ctx**	**6**	**42**	**0.023**
**Infralimbic ctx**	**0**	**19**	**0.033**	**Lateral posterior thalamic nucleus**	**8**	**45**	**0.013**	**Prelimbic ctx**	**5**	**12**	**0.026**
**Ventromedial thalamic nucleus**	**0**	**11**	**0.033**	**Periaqueductal gray thalamus**	**9**	**75**	**0.013**	**Medial pretectal area**	**0**	**0**	**0.028**
**Granular cell layer**	**22**	**85**	**0.034**	**Accumbens core**	**0**	**2**	**0.013**	**Lateral preoptic area**	**0**	**6**	**0.034**
**Accumbens shell**	**0**	**12**	**0.034**	**Medial dorsal thalamic nucleus**	**2**	**11**	**0.019**	**Anterior cingulate area**	**12**	**59**	**0.034**
**Triangular septal nucleus**	**1**	**5**	**0.034**	**Dentate gyrus ventral**	**4**	**24**	**0.019**	**Lateral septal nucleus**	**16**	**40**	**0.034**
**Tenia tecta ctx**	**6**	**46**	**0.034**	**Prelimbic ctx**	**1**	**18**	**0.023**	**Magnocellular preoptic nucleus**	**0**	**2**	**0.035**
**Cortical amygdaloid nucleus**	**11**	**26**	**0.037**	**Lateral septal nucleus**	**16**	**45**	**0.024**	**Accumbens core**	**0**	**2**	**0.039**
**Diagonal band of broca**	**0**	**4**	**0.038**	**Bed nucleus stria terminalis**	**2**	**14**	**0.026**	**Anterior hypothalamic area**	**2**	**10**	**0.049**
**Lateral preoptic area**	**0**	**7**	**0.039**	**Diagonal band of Broca**	**0**	**7**	**0.026**	Primary somatosensory ctx hindlimb	5	24	0.051
**3rd cerebellar lobule**	**2**	**7**	**0.04**	**Medial pretectal area**	**0**	**0**	**0.028**	Anterior pretectal nucleus	9	19	0.051
**Medial orbital ctx**	**0**	**15**	**0.042**	**Tenia tecta ctx**	**8**	**47**	**0.029**	Habenula nucleus	4	21	0.051
**Medial septum**	**0**	**1**	**0.044**	**Posterior hypothalamic area**	**0**	**18**	**0.03**	Lateral posterior thalamic nucleus	23	39	0.052
**Reuniens nucleus**	**0**	**2**	**0.044**	**Anterior cingulate area**	**4**	**50**	**0.03**	Ventral subiculum	19	50	0.052
Central medial thalamic nucleus	0	1	0.052	**Secondary somatosensory ctx**	**4**	**35**	**0.032**	Posterior hypothalamic area	2	15	0.053
Raphe linear	0	1	0.052	**Frontal association ctx**	**0**	**7**	**0.032**	3rd cerebellar lobule	7	15	0.057
Dorsal raphe	0	4	0.052	**Reuniens nucleus**	**0**	**3**	**0.033**	Periaqueductal gray thalamus	24	48	0.058
Lateral posterior thalamic nucleus	2	33	0.057	**Primary somatosensory ctx trunk**	**0**	**10**	**0.033**	Diagonal band of Broca	0	7	0.063
Primary somatosensory ctx trunk	0	10	0.058	**Magnocellular preoptic nucleus**	**0**	**1**	**0.035**	Extended amydala	0	2	0.063
Zona incerta	0	16	0.058	**Central gray**	**2**	**7**	**0.037**	Frontal association ctx	0	8	0.064
Primary somatosensory ctx upper lip	0	59	0.059	**Parafascicular thalamic nucleus**	**2**	**21**	**0.037**	Medial dorsal thalamic nucleus	3	10	0.069
Dentate gyrus ventral	1	8	0.061	**External plexiform layer**	**29**	**43**	**0.038**	Medial amygdaloid nucleus	5	14	0.069
Anterior hypothalamic area	0	17	0.065	**Glomerular layer**	**23**	**92**	**0.038**	Dentate gyrus ventral	5	15	0.069
Intercalated amygdaloid nucleus	0	0	0.067	**Ventromedial thalamic nucleus**	**0**	**6**	**0.045**	Ventrolateral thalamic nucleus	0	8	0.071
Medial pretectal area	0	0	0.067	**Premammillary nucleus**	**2**	**4**	**0.048**	Cortical amygdaloid nucleus	11	24	0.077
Substantia nigra reticularis	0	24	0.072	**Dorsomedial tegmental area**	**0**	**5**	**0.05**	Reuniens nucleus	0	3	0.081
Premammillary nucleus	2	4	0.073	**Primary somatosensory ctx hindlimb**	**0**	**43**	**0.05**	Secondary motor ctx	39	53	0.084

*Shown are three sets of results each representing comparisons between vehicle (Veh, n = 9) and idalopirdinen/donepezil (I/D, n = 9) treatment groups at times 15–25 (left), 25–35 (middle), and 35–45 (right) min post treatment. The probability values presented on the far-right column were derived using a Kruskall-Wallis test statistic. Lists were generated to include all areas (out of a total list of 171 areas that comprise the rat MRI atlas) that differed significantly (p < 0.05) for the epoch in which we observed the most changes (i.e., the 25–35 min epoch). Areas are rank-ordered by p-value for visualization. Areas highlighted in bold differed significantly between treatment groups. Areas listed in gray show areas that come closest to threshold for statistical significance*.

**Figure 2:**
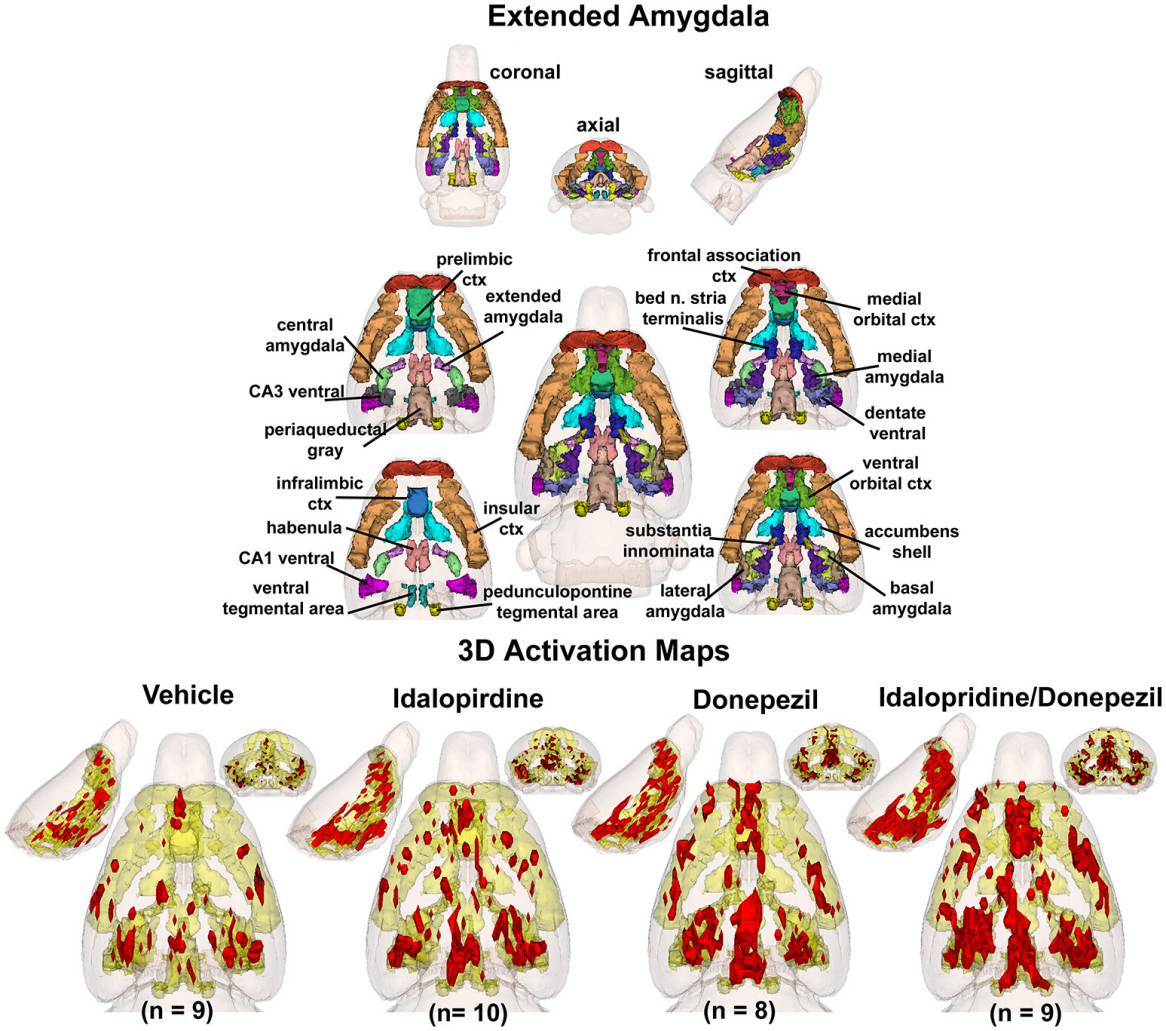
Extended-amygdala system. The 3D color model at the top depicts the location of 22 brain areas in the rat comprising the extended-amygdala system. These areas have been coalesced into a single volume (yellow) as shown in the lower 3D images for treatment groups, vehicle, idalopirdine, donepezil, and idalopirdine combined with donepezil. Areas in red are the localization of the activated voxels comprising the composite average from the rats (parentheses) in each experimental group. Once fully registered and segmented, the statistical responses for each animal are averaged on a voxel-by-voxel basis. Those averaged voxels that are significantly different from the 5 min baseline for positive BOLD in the 25–35 min post-treatment time window are show in their appropriate spatial location.

**Figure 3:**
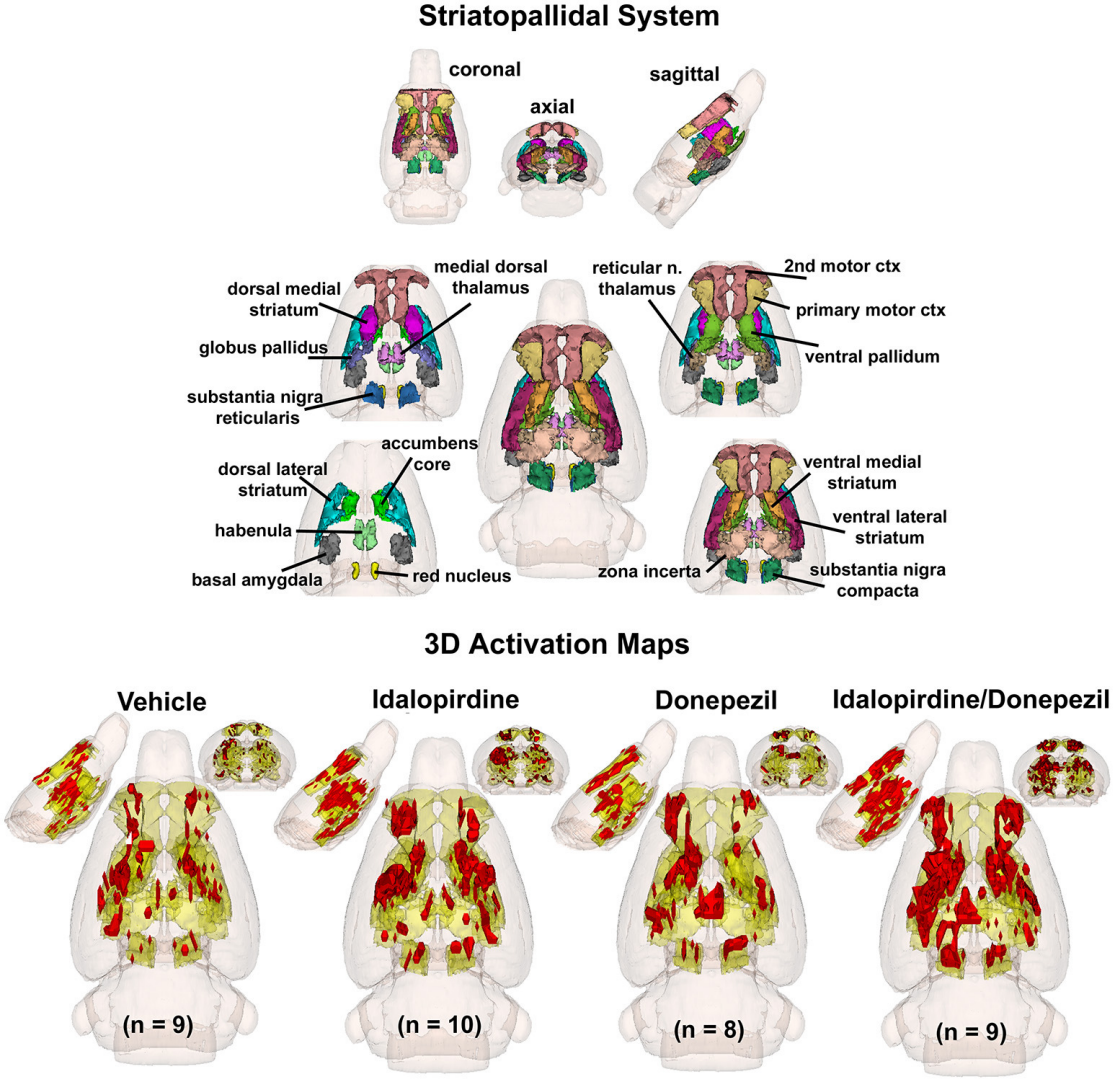
Striato-pallidal system. The 3D color model at the top depicts the location of 17 brain areas in the rat comprising the striato-pallidal system. Same as Figure 2.

**Figure 4:**
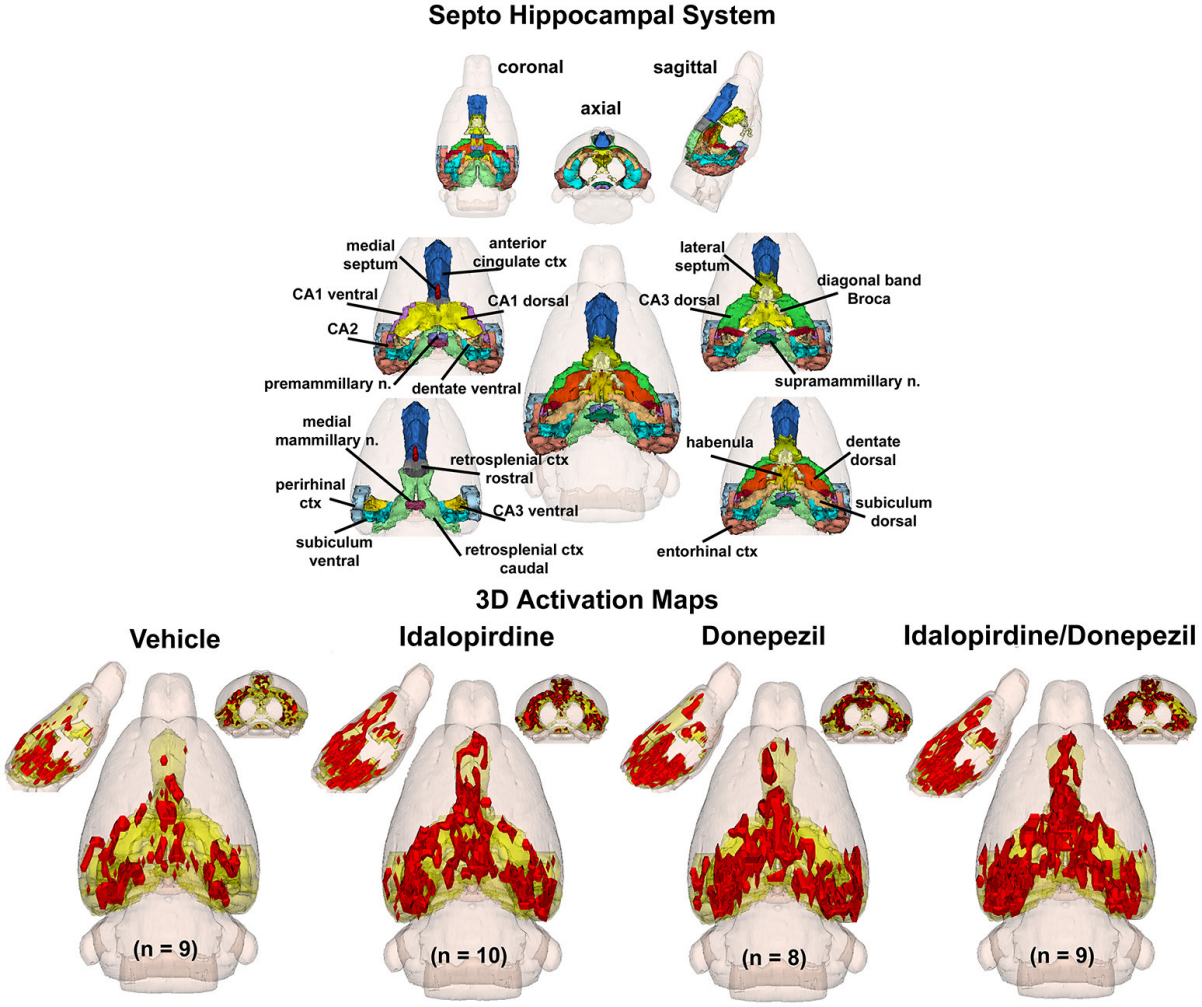
Septo-hippocampal system. The 3D color model at the top depicts the location of 21 brain areas in the rat comprising the septo-hippocampal system. Same as Figure 2.

**Figure 5:**
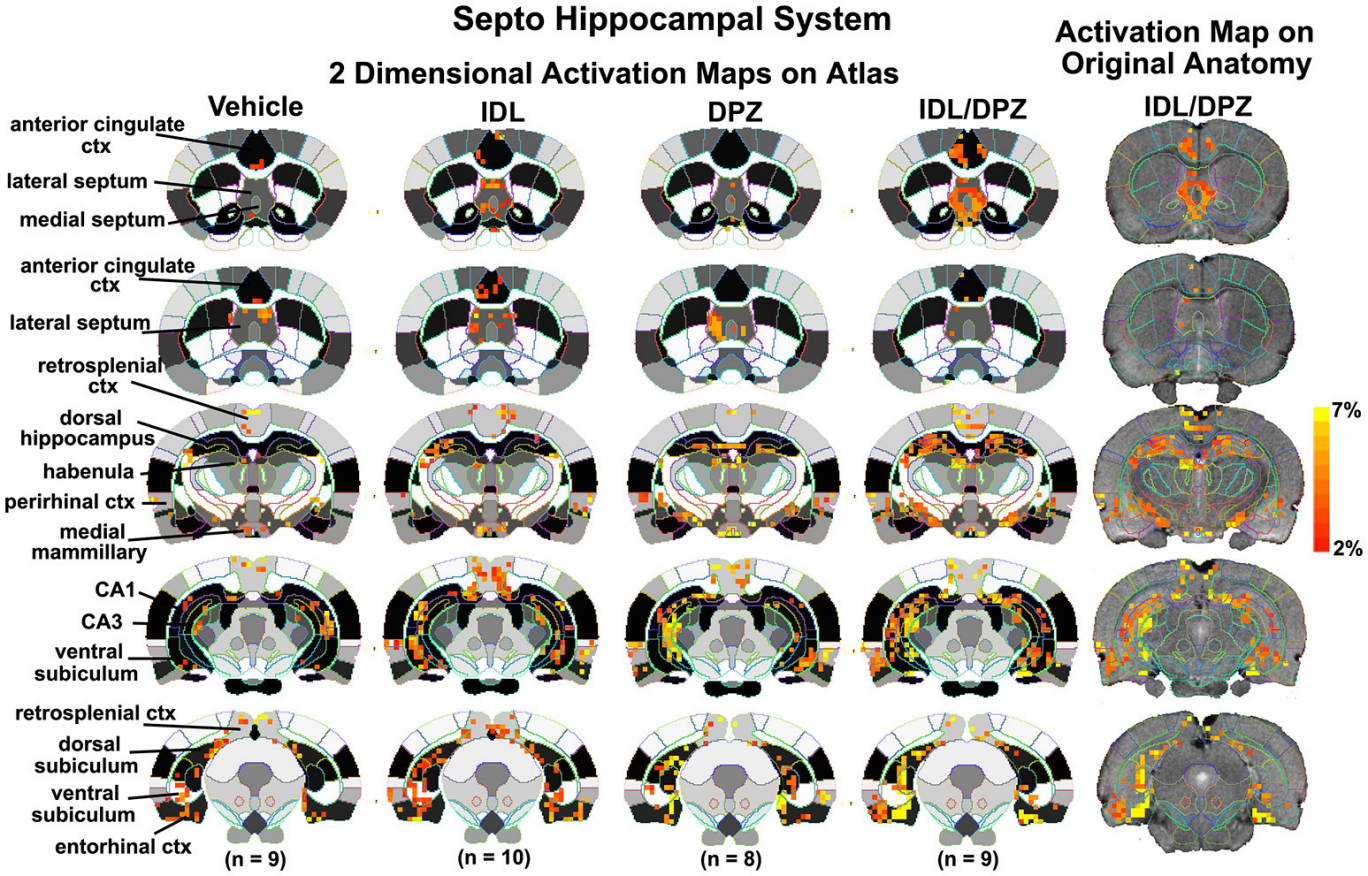
Two dimensional activation maps of the septo-hippocampal system. 2D activation maps from the rat brain atlas showing the precise location of the significantly altered positive (red) voxels for each of the experimental conditions taken from the 25 to 35 min post-treatment time window in the septo-hippocampal system. The figures on the right show the localization of the voxels on the original neuroanatomical images for the combined donepezil/idalopirdine condition. The vertical color strip indicates the percent change in BOLD signal. IDL, idalopirdine; DPZ, donepezil.

To better organize and visualize the many brain regions and signaling pathways activated by idalopridine and donepezil alone and in combination, as reported in Tables 1–3, three integrated neural circuits were defined—the extended-amygdala, striato-pallidal, and septo-hippocampal systems as shown in Figures 2–4 (Alheid, 2003). These circuits represent three major cortical-subcortical systems, although it is recognized that they are functionally intertwined and that various brain regions could be included in many different circuits. Figures 2–4 represent the 3D activation maps for each experimental condition (vehicle, idalopirdine alone, donepezil alone, idalopirdine, and donepezil in combination), taken from the 25 to 35 min post-treatment time window. These images give an indication of the global changes in the networks, whereas the statistical comparisons to vehicle treatment are to be drawn from Tables 1–3. In Figure 2, the 22 3D brain volumes comprising the extended-amygdala system are color coded and labeled as shown. These different brain regions are coalesced into a single volume (yellow/gold) below showing the localization of the average, significant volume of activation (red) for each experiment condition. This presentation of 3D data in 3D space clearly shows a robust activation of the extended-amygdala system in response to the combination treatment including, but not limited to, the brain regions that were activated by idalopirdine and donepezil alone. Whereas the pattern of activity is fairly similar in response to idalopirdine and donepezil alone (donepezil showing greater activation in the midline hindbrain regions, idalopirdine in the ventral CA1 and lateral amygdala), the combination treatment shows greater activation in the amygdala, nucleus accumbens, prelimbic, and infralimbic areas as well as recruitment of the PPT and the BNST. Shown in Figure 3 are the seventeen 3D brain regions that comprise the striato-pallidal system. For idalopirdine, activation of striatal regions and primary motor cortex stand out, for donepezil it is activation of the habenula. As in Figure 2, the combination treatment shows a greater volume of activation as compared to the other experiment conditions, with robust activation in the basal ganglia (substantia nigra, ventral pallidum, striatum, globus pallidus) and primary and secondary motor cortices. Shown in Figure 4 are the 20 3D brain regions that comprise the septo-hippocampal system. In addition, the activation pattern in the septo-hippocampal system is represented as 2D data in 2D space (Figure 5), with the actual location of the average significantly activated voxels registered to the rat atlas and in their position in original raw anatomy. Looking across the first row of axial images, note the heightened activation of the septal area with the combination treatment. Again, the pattern of activation with the combination treatment exceeds that of either idalopirdine or donepezil alone and this pattern of synergistic activity continues through the hippocampal complex as seen in the lower rows of more caudal axial sections. For example, combining the voxels numbers for dorsal and ventral CA3 (see Supplementary Tables 1–3) shows vehicle with 14 voxels, donepezil with 26 voxels, idalopirdine with 24 voxels and the combination of both with 48 voxels. This pattern toward higher activation with the combination treatment and the location of the activated voxels are visualized in the 2D activation maps (Figure 5). However, it should be noted that the hippocampal complex shows heightened activity for all of the four experiment conditions and, while the combination treatment shows the highest volume of activation (voxels) by two to three fold, it does not reach significance in the statistical analysis when compared to vehicle treatment (Table 3).

### Comparison of the treatments to vehicle and to each other for bold signal over time

**Figure 6:**
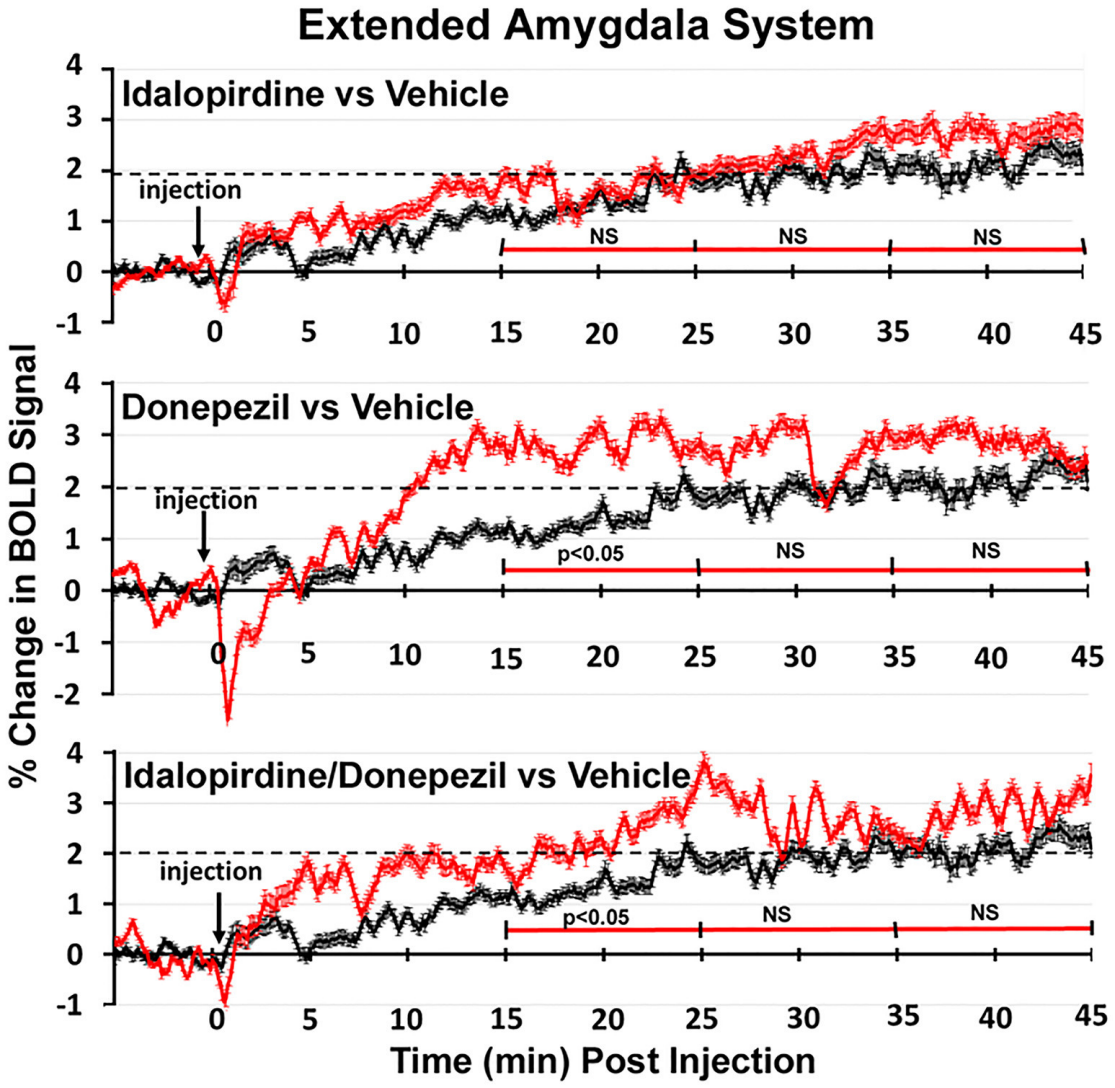
Time course plots for BOLD in the extended-amygdala system. Shown are the changes in BOLD signal (red) over 45 min (450 image acquisitions) for each of the different drug treatments as compared to vehicle (black). Each of the 450 time points (drug and vehicle) are the mean of all brain areas in the extended-amygdala system (see Figure 2). The red time line is segmented into the periods reported in the Tables and show the significant differences between drug and vehicle at each period. Vertical bars denote SEM.

**Figure 7:**
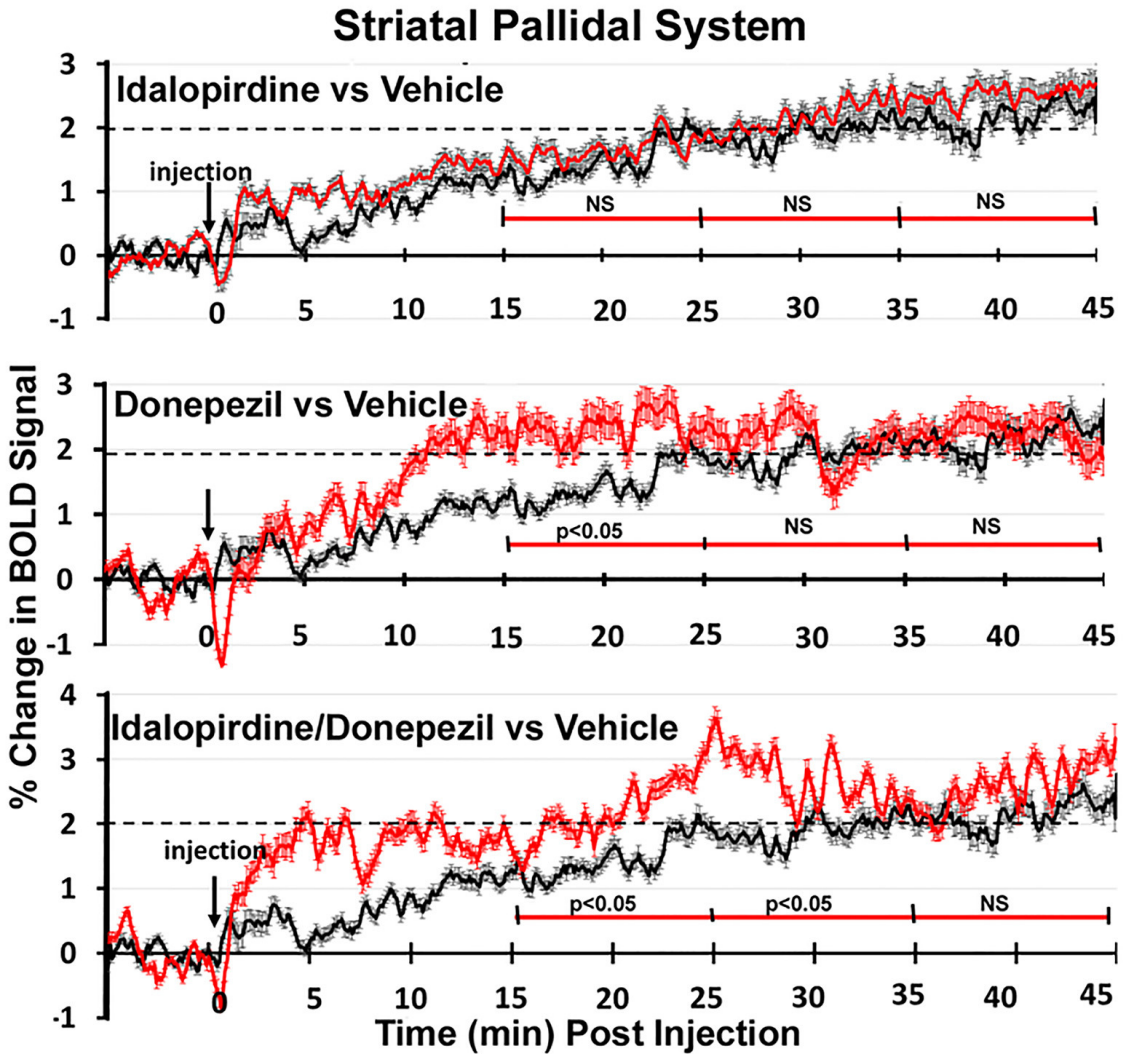
Time course plots for BOLD in the striato-pallidal system. Same as Figure 6.

**Figure 8:**
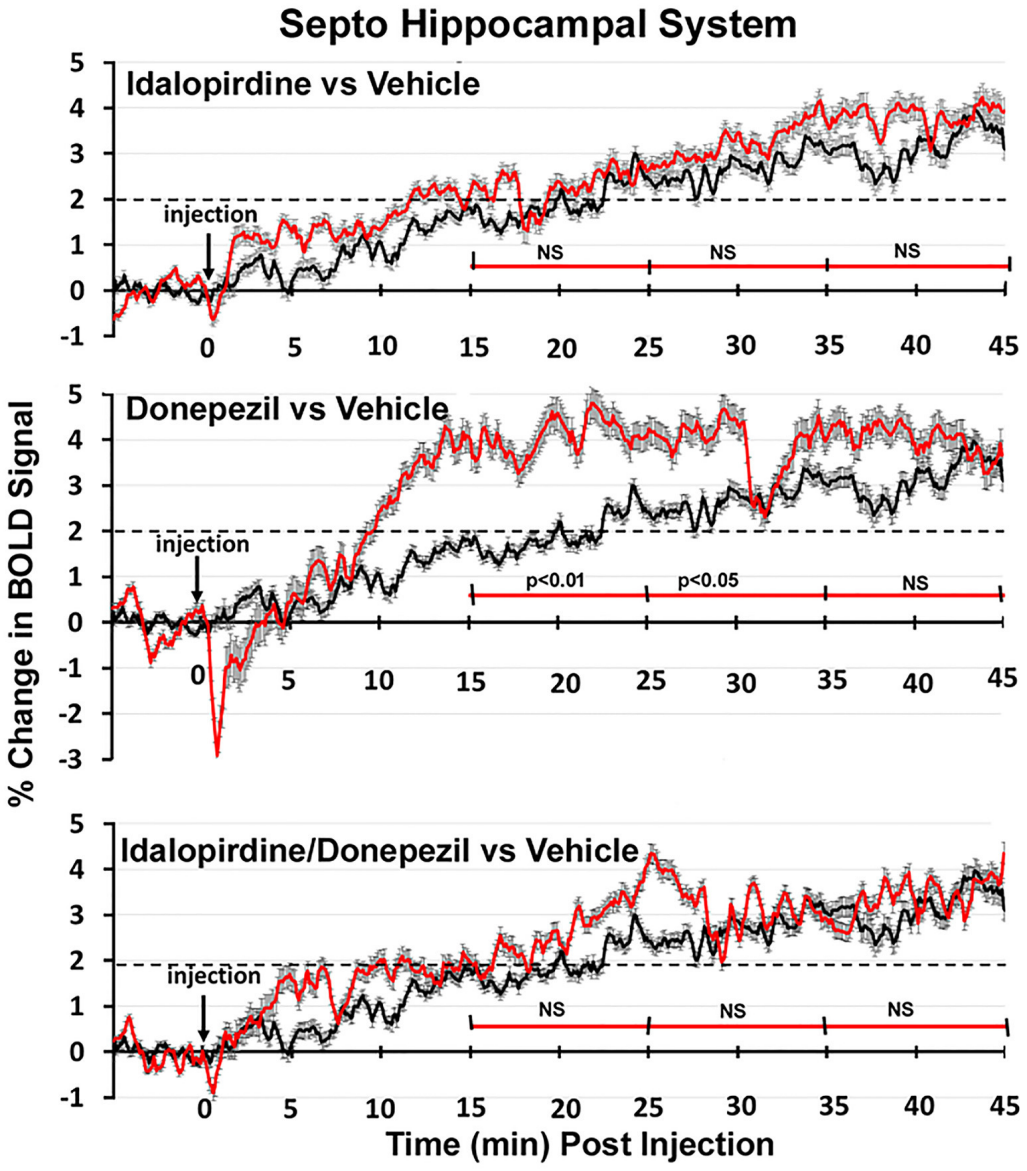
Time course plots for BOLD in the septo-hippocampal system. Same as Figure 6.

Shown in Figure 6 are the changes in BOLD signal over the 50 min imaging session for each drug treatment versus vehicle for the extended-amygdala system. A repeated measures ANOVA showed a significant interaction between treatments over time [*F*_(3, 147)_ = 1.35; *p* < 0.0001]. Dunnett's *post-hoc* analysis at 15–25 min showed a significant difference only between the combination of idalopirdine plus donepezil vs. vehicle (*p* < 0.05). There were no significant differences for any of the treatments at times 25–35 and 35–45. Figure 7 shows the change in BOLD signal for each treatment in the striato-pallidal system. There was a statistical trend for the interaction between treatments over time [*F*_(3, 144)_ = 1.20, *p* < 0.063]. Dunnett's *post-hoc* analysis at 25–35 min showed a statistical trend for the difference between the combination of idalopirdine plus donepezil vs. vehicle (*p* < 0.10). There were no significant differences for any of the treatments at other timepoints. Figure 8 shows the change in BOLD signal for each treatment in the septo-hippocampal system. There was a significant interaction between treatments [*F*_(3, 144)_ = 1.73; *p* < 0.012]. Dunnett's *post-hoc* analysis at 15–25 min showed a significant difference between donepezil vs. vehicle (*p* < 0.05). There were no significant differences for any of the treatments at other timepoints.

## Discussion

Studies were performed to evaluate how idalopirdine, donepezil, and the combination of both affect brain activity as measured by BOLD MRI in awake rats. A discrete number of brain regions were significantly activated by both compounds individually, suggesting that they share recruitment of the forebrain cholinergic system (dBB), the infralimbic cortex, the ventral pallidum, the nucleus accumbens shell, and the magnocellular preoptic area. In addition, donepezil activated a number of cortical regions, the serotonergic dorsal raphe nucleus, and areas within the septo-hippocampal system, though remarkably not the hippocampal complex itself. When both treatments were combined, there were clear synergistic effects on brain activity, with activation of additional regions within the extended-amygdala, striato-pallidal and septo-hippocampal systems and of the cholinergic PPT. In addition, idalopirdine, alone or in combination with donepezil, may modulate arousal from exteroceptive (olfactory system) and interoceptive (brainstem) stimuli. Collectively, these data show that the synergistic activity of idalopirdine and donepezil is spread across at least three integrated neural networks and includes further activation of the cholinergic system. The functional implications of activating the brain regions and circuits identified in the current study remain to be established. In the following paragraphs, we discuss the potential relevance of these findings with emphasis on the procognitive effects of idalopirdine, donepezil, and the combination of both.

### Effects of idalopirdine

When reviewing the data from all 171 brain regions for significant changes in volume of activity and the BOLD signal change over time in the three neuronal circuits, few brain regions were significantly activated by idalopirdine alone in comparison to vehicle or the other drug treatments. Of the regions with high-moderate 5-HT_6_ receptor expression (striatal complex, olfactory bulb, hippocampus, cortex), only the nucleus accumbens shell (ventral striatum) and the infralimbic cortex were significantly activated. This may indicate that, under these imaging conditions, there is insufficient serotonergic tone on the 5-HT_6_ receptor and/or engagement of the associated neuronal circuits to generate a signal. However, comparison of these results with donepezil alone and the combined treatment suggests a more complicated set of biological interactions. Indeed, previous studies have shown that some effects of 5-HT_6_ receptor antagonism require the presence of an AChEI (Dawson, 2011; Herrik et al., 2016). The mechanisms underlying the functional interaction between an AChEI and a 5-HT_6_ receptor antagonist are poorly understood, but the current observation that donepezil activates the dorsal raphe nucleus may indicate that this AChEI can enhance serotonergic tone. When comparing the combination treatment to donepezil alone (Supplementary Table 6), we did observe activation of 5-HT_6_ receptor-expressing regions, including multiple striatal regions and the hippocampal CA2 area, indicating that addition of idalopirdine to donepezil does result in engagement of brain regions with high 5-HT_6_ receptor expression, which is also supported by the 2 and 3D images of the striato-pallidal and septo-hippocampal systems.

### Effects of donepezil

Donepezil alone activated multiple regions of the limbic cortex and the septo-hippocampal system (medial septum, ventral dentate gyrus, and entorhinal cortex), and increased BOLD signal in all three neuronal circuits in discrete time periods, an effect which was most pronounced in the septo-hippocampal system. These observations fit well with the mechanism of action and the procognitive profile of the compound. Indeed, the basal forebrain cholinergic system, originating in the medial septum, and the dBB, provides cholinergic innervation to the hippocampus and cortical regions. Donepezil activated both forebrain cholinergic nuclei in the current study and, through inhibition of AChEI, increases the levels of the excitatory neurotransmitter ACh in the terminal regions. Remarkably though, the hippocampal complex itself was not significantly activated by donepezil in the present study, which is discussed in more detail below.

### Effects of combination treatment

#### The cholinergic system

The combination treatment activated the PPT, part of the brain-stem cholinergic system, in addition to the basal forebrain cholinergic nuclei (medial septum and dBB) that were also activated by donepezil alone or both compounds individually. Electrical stimulation of the PPT has been demonstrated to increase cortical arousal (Dringenberg and Olmstead, 2003), enhance cortical acetylcholine release (Rasmusson et al., 1994) and facilitate conditioned learning (Andero et al., 2007). The current finding that the combination treatment activates the PPT, supports our previous observations that idalopirdine potentiates the effects of donepezil on cortical extracellular ACh levels and excitability, manifested as gamma oscillations in the EEG (Amat-Foraster et al., 2016). Indeed, activation of the PPT enhances gamma oscillatory activity in the cortex through stimulation of thalamocortical projections (Steriade, 2006).

The PPT (or pendunculopontine nucleus, PPN) also plays a role in the regulation of gait and postural stability and loss of cholinergic neurons in this region is thought to contribute to deficits in these functions in Parkinson's disease (PD), parasupranuclear palsy (PSP), and multi systems atrophy (MSA; Bohnen and Albin, 2011; Benarroch, 2013). Consistent with this possibility, the severity of cholinergic neuronal loss in the PPN correlates with the severity of parkinsonian symptoms in PD and lesions involving the PPN manifest with gait disturbances (Aziz et al., 1998; Rinne et al., 2008). We have recently demonstrated that the combination of idalopirdine and donepezil reduces falls in an animal model of PD with dual dopaminergic and cholinergic lesions (Kucinski et al., 2017). This, together with the activation of the PPT in the current study, would suggest that the combination treatment might have beneficial effects on gait and posture through activation of the brainstem cholinergic system.

#### The extended-amygdala system

The dopaminergic projections from the VTA to the nucleus accumbens shell and limbic cortex (including the infralimbic and prelimbic cortices) play a critical role in reward and motivation (Koob, 1996; Malenka et al., 2009). These, as well as other regions included in the extended-amygdala system in the current analysis (several amygdaloid nuclei, BNST, habenula), were activated by the combination treatment, which also significantly increased the BOLD signal as compared to vehicle treatment between 15 and 25 min after administration. These observations suggest an important role for the extended-amygdala system in the combined effects of idalopirdine and donepezil. Indeed, there is ample evidence that reward and emotional arousal, in which the amygdala plays a central role, contribute to both memory encoding and consolidation (McGaugh et al., 1996; Phelps and Anderson, 1997; Miendlarzewska et al., 2016). The amygdala also plays an important role in the regulation of anxiety and fear. Interestingly, a few studies have suggested that 5-HT_6_ receptor antagonists may reduce anxiety and depressive-like behavior in rodent models (Wesolowska and Nikiforuk, 2007; Wesolowska and Jastrzebska-Wiesek, 2011), although the combined effects of 5-HT_6_ antagonists with AChEIs have not been tested. When regarding the volume of activation, as outlined in Tables 2, 3, a number of brain regions were activated to a greater extent with the combination treatment as compared to donepezil alone.

#### The striato-pallidal system

Of the regions included in the striato-pallidal system, few were significantly activated by the individual treatments, with the exception of the substantia nigra pars reticularis by idalopirdine and the ventral pallidum by both treatments individually. The combination treatment, in comparison to vehicle, activated additional regions within this system but, as described before, not the striatal complex itself. However, when the combination treatment was compared to donepezil alone (see Supplementary Table 6), activation of multiple striatal regions, additional thalamic nuclei as well as the globus pallidus was observed, which is supported by the 3D activation maps of the striato-pallidal system and the more robust increase in BOLD signal over time as observed with the combination treatment in the striato-pallidal system as a whole.

Recruitment of the striatal-pallidal system may contribute to the procognitive effects of idalopirdine when combined with donepezil. The ventral striatum, in particular the nucleus accumbens shell, mediates cognition related to reward, reinforcement, and motivational salience as discussed above. The dorsal striatum mediates cognition involving stimulus-response learning, motor function and certain executive functions (Self and Nestler, 1995; Devan et al., 2011; Daniel and Pollmann, 2014). Within the dorsal striatum, the dorsal medial sub-region mediates goal-directed learning whereas the dorsal lateral striatum contributes to the acquisition of habits (Yin et al., 2004, 2005a,b; Balleine and O'Doherty, 2010; Liljeholm and O'Doherty, 2012; Burton et al., 2015).

The striato-pallidal system also plays a crucial role in regulation of movement, again depending on the striatal sub-region (Ohno et al., 2011). The current observation that addition of idalopirdine to donepezil results in activation of the dorsal lateral striatum, nucleus accumbens core, globus pallidus, and connected thalamic nuclei, when compared to donepezil alone, suggests engagement of the motor circuitry by the combination treatment. This is further supported by the enhanced signal in the primary and secondary motor cortices with the combination treatment in the 3D activation map of the striato-pallidal system.

It is conceivable that activation of the dopaminergic midbrain system, specifically the elements that integrate the nigrostriatal pathway, may be related to the factor of restriction of movement that the rats underwent during the experimental phase, despite the fact that all treatment groups were handled equally. This cannot be addressed with the current technology as restriction of movement is a prerequisite for the imaging procedure.

#### The septo-hippocampal system

The combination treatment activated several additional regions within the septo-hippocampal system (most notably in the septum), when compared to the effects of donepezil alone. However, neither donepezil nor the combination treatment significantly activated the hippocampal complex itself when compared to vehicle treatment. This came as a surprise, given the vast literature on the hippocampus, learning and memory, and the fact that AChEIs were shown to activate the hippocampus and improve hippocampal network connectivity in AD patients (Goekoop et al., 2006; Goveas et al., 2011). However, the cholinergic medial septum/dBB complex, a key modulator of hippocampal activity and rhythmogenesis, was activated by donepezil and the combination treatment in the current study (Dannenberg et al., 2015). Furthermore, we have recently demonstrated that donepezil increases hippocampal theta and gamma oscillations during electrical brainstem stimulation in the anesthetized rat, an effect which was further potentiated by idalopirdine (Herrik et al., 2016). The apparent discrepancy between this and the current study may be explained by a different level of engagement of the hippocampal formation. In the study by Herrik et al., electrical stimulation of the reticular formation provides heightened afferent input to the hippocampus, including enhanced cholinergic innervation (Herrik et al., 2016), whereas in the current study the rats, though awake, did not receive any salient or cognitively demanding cues. Under the conditions of the fMRI, there may simply not be enough afferent drive to reveal significant effects of donepezil and the combination treatment on hippocampal activity. In addition, the current study was performed in young, healthy rats and not in an AD-relevant disease model. The data would suggest the combination treatment engages discrete regions of the septo-hippocampal system which plays a critical role in learning and memory (Morris et al., 1982; Eichenbaum, 2000; Burgess et al., 2002; Lecourtier et al., 2011), but that these effects are not as pronounced as in the extended-amygdala and striato-pallidal systems under the current conditions of fMRI imaging.

A remarkable finding is that both donepezil and the combination treatment induced widespread activation in the entorhinal cortex, the region where AD pathology starts and which is considered a gate-keeper for the subsequent spread of AD pathology to the hippocampal formation and cortical regions (Braak and Braak, 1991; Van Hoesen et al., 1991; Khan et al., 2014). In addition, the combination treatment activated the anterior cingulate cortex, a region which has been demonstrated to have reduced metabolism in AD patients with apathy—the most common neuropsychiatric symptom and a symptom associated with worse prognosis for cognitive and functional progression (Marshall et al., 2007; Guimaraes et al., 2008; Stanton et al., 2013; Stella et al., 2014). By increasing activity in the entorhinal and anterior cingulate cortices, the combination treatment may enhance function of these brain regions affected during the course of AD pathology.

While the drugs included in this study may have effects on peripheral autonomic physiology, especially in the case of the AChEI Donepezil, coordination between blood flow and neuronal activity within the brain parenchyma is tightly regulated due to the high metabolic demand of neuronal tissue. Thus, it is doubtful that the drug treatments in this study produced their effects via direct modulation of vascular responses within the brain parenchyma. However, it is possible that drug treatments modulated peripheral physiology (e.g., noradrenaline activity) thereby initiating an interoceptive cascade that ultimately resulted in BOLD changes in the brain. Future studies are required to determine whether the drug treatments employed in this study act through modulation of peripheral physiology.

### Caveats

For any imaging study on awake animals the issues and consequences related to the stress of head restraint and restricted body movement must be considered. Protocols have been developed to help lessen the stress of an imaging study by acclimating animals to the environment of the MR scanner and the restraining devices helping to reduce stress hormones levels and measures of sympathetic autonomic activity (Zhang et al., 2000; King et al., 2005). These acclimation procedures put animals through several simulated imaging sessions and have been used to study sexual arousal in monkeys (Ferris et al., 2004), generalized seizures in rats and monkeys (Tenney et al., 2003), and exposure to psychostimulants like cocaine (Febo et al., 2004, 2005; Ferris et al., 2005), nicotine (Skoubis et al., 2006), amphetamine (Madularu et al., 2015), and apomorphine (Zhang et al., 2000; Chin et al., 2006). Nonetheless, one must consider the experimental confound that exists with low levels of arousal and stress associated with imaging awake animals. In addition, one must consider the potential effect of earlier exposure to isoflurane during the set-up prior to imaging and the time that lapses between the final day of acclimation and imaging.

The two measures of BOLD signal change reported here, percent change in BOLD signal over time and volume of activation, clearly show the synergistic effect of idalopirdine plus donepezil on brain activity. The change in BOLD signal over time is the mean of all activated voxels from brain areas comprising the neuronal circuit of interest (e.g., 21 areas for the extended amygdala) while the volume of activation is the number of significantly activated voxels for each of 171 brain areas. Given the high level of multiple comparisons the latter method is open to the possibility of false positives and should be considered when focusing on any specific brain area.

This study provides insight into the pharmacological effects of donepezil, idalopirdine and the combination of both on brain activity in the awake rat. To further study the implications of these for AD in particular and cognitive impairment in general, further studies employing animal models capturing neuropathological features of AD and cognitive impairment are crucial.

## Conclusion

In summary, the current data indicate that, whilst idalopirdine and donepezil recruit a discrete number of overlapping brain regions including one of the forebrain cholinergic nuclei, the synergistic effect of combining treatment extends beyond the effects of donepezil alone and the cholinergic system, toward recruitment of multiple neural circuits and neurotransmitter systems. Indeed, the combination treatment recruits a constellation of integrated neural circuits associated with cognition, emotion and motivation as well as exteroceptive (olfaction) and introceptive cues (brainstem). These may collectively contribute to enhancing cognition, by enriching learning and memory processes with motivational salience and the context of extro- and intro-ception. These data provide new insight into how idalopirdine may extend and complement the benefits of donepezil observed in patients with AD (Wilkinson and Windfeld, 2014).

## Author contribution

All authors had full access to all the data in the study and take responsibility for the integrity of the data and the accuracy of the data analysis. Study concept and design: Id, CF, MN. Acquisition of data: CF, PK, MN. Analysis and interpretation of data: CF, PK, JY. Drafting of the manuscript: Id, CF, PK. Critical revision of the manuscript for important intellectual content: Id, PK, CF. Statistical analysis: PK, CF. Administrative, technical, and material support: PK, JY. Study supervision: Id, CF, MN. All authors agree to be accountable for all aspects of the work.

### Conflict of interest statement

CF, PK, and MN have employments at EKAM imaging. Id is a full-time employee at H. Lundbeck A/S.

## Abbreviations

5-HT_6_ receptor: 5-hydroxytryptophan 6 receptor
5-HT: 5-hydroxytryptophan (serotonin)
ACh: acetylcholine
AChEI: acetylcholinesterase inhibitor
AD: Alzheimer's disease
BNST: bed nucleus of the stria terminalis
BOLD: blood oxygen level dependent
dBB: diagonal band of Broca
EEG: electroencephalogram
fMRI: functional magnetic resonance imaging
FOV: field of vision
FWHM: Full Width Half Max
GABA: gamma amino butyric acid
i.v.: intravenous
MIVA: Medical Image Visualization and Analysis
MS/MS: tandem mass spectrometry
(m)PFC: (medial) prefrontal cortex
nAcc: nucleus accumbens
NEX: number of averages
nPO: nucleus pontis oralis
PAG: periaqueductal gray
p.o.: per oral
PPT: pedunculopontine tegmentum
RARE: rapid acquisition with relaxation enhancement
REM: rapid eye movement
s.c.: subcutaneous
SEM: standard error of means
TE: echo time
Ti-1: inverse transformation matrix
TR: repetition time.
