# Supplementary material for: The Serotonin Receptor 6 Antagonist Idalopirdine and Acetylcholinesterase Inhibitor Donepezil Have Synergistic Effects on Brain Activity—A Functional MRI Study in the Awake Rat
Source: Front Pharmacol. 2017 Jun 12;8:279. doi: 10.3389/fphar.2017.00279 (PMC5467007; doi:10.3389/fphar.2017.00279)

**Supplementary table 1: fMRI BOLD response for idalopirdine compared to vehicle following a single administration – total list of 171 brain regions.**

Shown in the middle columns are the median number of activated voxels following vehicle (Veh, n = 9) and idalopirdine (I, n = 10) treatment at times 15-25, 25-35 and 35-45 min post treatment. The probability values are presented on the far right column using a Wilcoxon rank-sum test statistic. The table displays all 171 brain regions that comprise the rat MRI atlas, ranked in order of their significance.


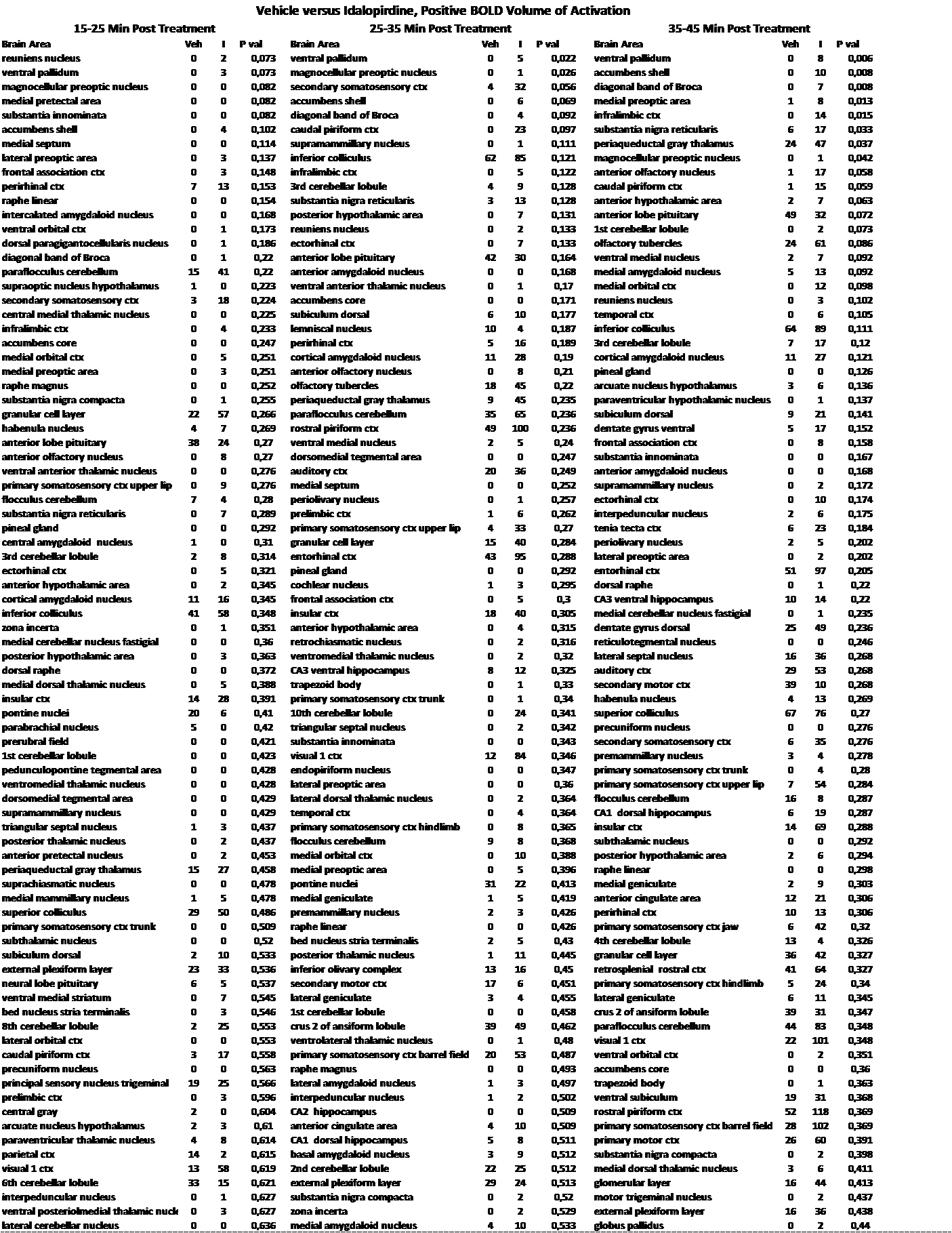


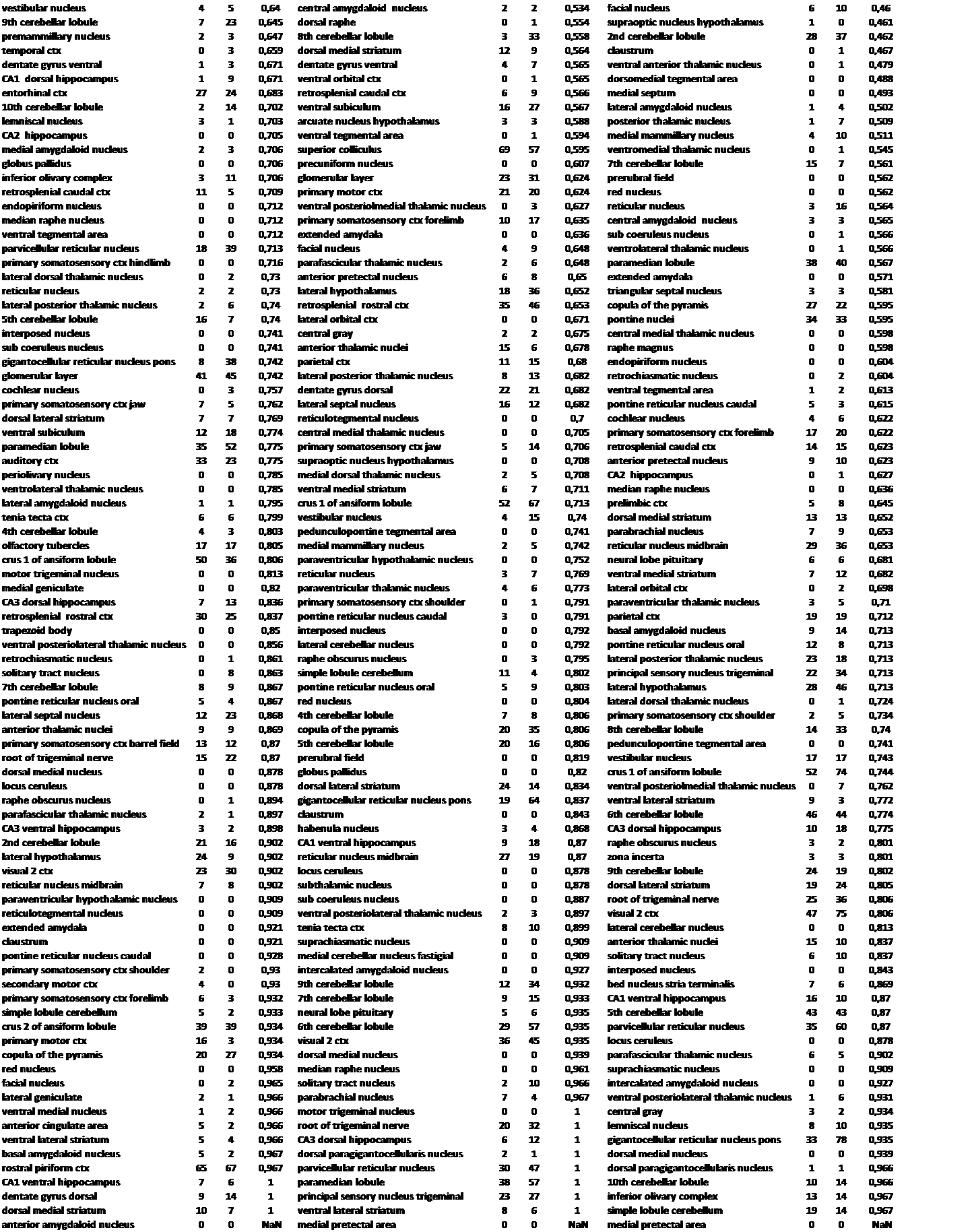

Supplement: Supplementary file 1 [file Table1.DOCX]
