# Supplementary material for: The Serotonin Receptor 6 Antagonist Idalopirdine and Acetylcholinesterase Inhibitor Donepezil Have Synergistic Effects on Brain Activity—A Functional MRI Study in the Awake Rat
Source: Front Pharmacol. 2017 Jun 12;8:279. doi: 10.3389/fphar.2017.00279 (PMC5467007; doi:10.3389/fphar.2017.00279)

**Supplementary table 5: fMRI BOLD response for the combination of donepezil plus idalopirdine compared to idalopirdine following a single administration.**

Shown in the middle columns are the median number of significantly activated voxels following idalopirdine (I, n = 10) and idalopirdine combined with donepezil (I/D, n = 9) at times 15-25, 25-35 and 35-45 min post treatment. See Table 1 legend.


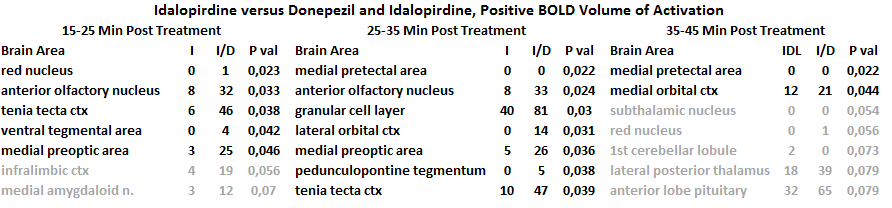

Supplement: Supplementary file 5 [file Table5.DOCX]
